# *Erwinia carotovora *elicitors and *Botrytis cinerea *activate defense responses in *Physcomitrella patens*

**DOI:** 10.1186/1471-2229-7-52

**Published:** 2007-10-08

**Authors:** Inés Ponce de León, Juan Pablo Oliver, Alexandra Castro, Carina Gaggero, Marcel Bentancor, Sabina Vidal

**Affiliations:** 1 Departamento de Biología Molecular, Instituto de Investigaciones Biológicas Clemente Estable, Avenida Italia 3318, CP 11600, Montevideo, Uruguay; 2 Laboratorio de Biología Molecular Vegetal, Facultad de Ciencias, Universidad de la República, Iguá 4225, CP 11400, Montevideo, Uruguay

## Abstract

**Background:**

Vascular plants respond to pathogens by activating a diverse array of defense mechanisms. Studies with these plants have provided a wealth of information on pathogen recognition, signal transduction and the activation of defense responses. However, very little is known about the infection and defense responses of the bryophyte, *Physcomitrella patens*, to well-studied phytopathogens. The purpose of this study was to determine: i) whether two representative broad host range pathogens, *Erwinia carotovora *ssp.* carotovora *(*E.c. carotovora*) and *Botrytis cinerea *(*B. cinerea*), could infect *Physcomitrella*, and ii) whether *B. cinerea*, elicitors of a harpin (HrpN) producing *E.c. carotovora *strain (SCC1) or a HrpN-negative strain (SCC3193), could cause disease symptoms and induce defense responses in *Physcomitrella*.

**Results:**

*B. cinerea *and *E.c. carotovora *were found to readily infect *Physcomitrella *gametophytic tissues and cause disease symptoms. Treatments with *B. cinerea *spores or cell-free culture filtrates from *E.c. carotovora*_SCC1 _(CF_(SCC1)_), resulted in disease development with severe maceration of *Physcomitrella *tissues, while CF_(SCC3193) _produced only mild maceration. Although increased cell death was observed with either the CFs or *B. cinerea*, the occurrence of cytoplasmic shrinkage was only visible in Evans blue stained protonemal cells treated with CF_(SCC1) _or inoculated with *B. cinerea*. Most cells showing cytoplasmic shrinkage accumulated autofluorescent compounds and brown chloroplasts were evident in a high proportion of these cells. CF treatments and *B. cinerea *inoculation induced the expression of the defense-related genes: *PR-1*, *PAL*, *CHS *and *LOX*.

**Conclusion:**

*B. cinerea *and *E.c. carotovora *elicitors induce a defense response in *Physcomitrella*, as evidenced by enhanced expression of conserved plant defense-related genes. Since cytoplasmic shrinkage is the most common morphological change observed in plant PCD, and that harpins and *B. cinerea *induce this type of cell death in vascular plants, our results suggest that *E.c. carotovora *CF_SCC1 _containing HrpN and *B. cinerea *could also induce this type of cell death in *Physcomitrella*. Our studies thus establish *Physcomitrella *as an experimental host for investigation of plant-pathogen interactions and *B. cinerea *and elicitors of *E.c. carotovora *as promising tools for understanding the mechanisms involved in defense responses and in pathogen-mediated cell death in this simple land plant.

## Background

Plants are continuously subjected to pathogen attack and respond by activating a range of defense mechanisms. Recognition of the pathogen or elicitors derived either from the pathogen or released from the plant cell wall is accompanied with the production of molecular signals including salicylic acid [1], jasmonic acid [2] and ethylene [3] that lead to the induction of defense gene expression. This in turn results in the accumulation of functionally diverse pathogenesis-related (PR) proteins and metabolites (*e.g*., phenylpropanoids) [4,5]. Recognition of the pathogen or elicitors is usually accompanied by the rapid death of the infected cells, known as the hypersensitive response (HR), which limits the access of the pathogen to water and nutrients thereby restricting its growth [6,7]. HR can be triggered either by non-specific elicitors recognized by plant receptors, or by specific elicitors (encoded by pathogen avirulence (*avr*) genes) recognized by corresponding encoded products of plant resistance (*R*) genes [8,9]. Several studies have suggested that plant cell death resulting from the HR is a type of programmed cell death (PCD). Plant cells undergoing PCD share a number of characteristic morphological and biochemical features in common with animal cell apoptosis [7,10,11]. Moreover, cell death with apoptotic features has also been observed in plants susceptible to virulent pathogens [12,13].

Although bryophytes are non-vascular plants and are considered to be primitive among the embryophyta, mosses have been shown to respond to a variety of environmental stimuli and to several common plant growth factors much like vascular plants. Thus, in spite of having diverged from vascular plants approximately 700 million years ago [14], mosses are well-suited for the study of fundamental processes in plant biology. Furthermore, mosses have a simple developmental program and a life cycle with a predominant haploid phase which greatly facilitates genetic analysis [15].

*Physcomitrella patens*, a relatively small moss, has recently become a model plant to study plant gene function in that it exhibits high-frequency homologous recombination comparable with that of *Saccharomyces cerevisiae*, enabling the construction of gene knock-outs [16,17]. The assembled *Physcomitrella *genome has recently been released and full-length cDNAs in addition to 80,000 ESTs are available in the databases [18-20]. These advantages together with the presence of a great number of *Physcomitrella *ESTs with high sequence identity to defense-related genes of vascular plants, many of them with unknown functions, makes this plant a very useful model to study plant-pathogen interactions. The susceptibility of distinct tissues to pathogens can also be studied, since *Physcomitrella *can be maintained as a haploid gametophyte with distinct developmental stages. These consist of the protonema which is a filamentous network of cells, and the radially symmetric gametophore which is a leafy shoot composed of a non-vascular stem with leaves as well as rhizoids [21]. Disease development can be visualized microscopically in that the leaves and protonemal filaments are formed of a monolayer of cells.

There have been very few reports on either pathogen infection or the activation of defense responses in mosses. *In silico *analysis of the *Physcomitrella *genome, however, indicates the presence of several encoded proteins with high similarity to *R *gene products found in flowering plants [22]. Regarding natural infection, the fungus *Scleroconidioma sphagnicola *(*S. sphagnicola*) can infect and cause disease symptoms in the moss *Sphagnum fuscum *(*S. fuscum*) [23] and viruses were detected in Antarctic mosses [24]. *S. sphagnicola *hyphae can grow inside the cell wall of *S. fuscum*, digesting wall components, penetrating into cells of leaves and causing chlorosis of the tissue. In more advanced stages of disease development, necrosis of infected leaf and stem cells, as well as host death can be observed [23].

In this study we aimed to identify plant pathogens capable of infecting and triggering a defense response in *Physcomitrella*, with the goal of establishing a model system to conduct molecular, cellular and genetic studies on *Physcomitrella*-pathogen interactions. We used two pathogens with a broad host range, the bacterium *Erwinia carotovora *ssp *carotovora *(*E.c. carotovora*) and the fungus *Botrytis cinerea *(*B. cinerea*). *E.c. carotovora *is a soft-rot *Erwinia *which causes disease on many vascular plants [25,26]. The main virulence factors of *E.c. carotovora *are the plant cell wall-degrading enzymes including cellulases, proteases and pectinases [26]. These enzymes cause maceration of the infected tissues and the released cell wall fragments can act as elicitors of the plant defense response [27-31]. Previous studies have shown that cell-free culture filtrate (CF) containing plant cell wall-degrading enzymes from *E.c. carotovora*_SCC3193 _produces similar symptoms and defense gene expression as those caused by *E.c. carotovora*_SCC3193 _infection and enhanced disease resistance in CF-treated plants [28,30-32]. Some *E.c. carotovora *strains produce harpins, which are small, acidic, glycine-rich, heat-stable proteins, that elicit HR and induction of plant defense responses [33,34]. In the present study we have used two strains of *E.c. carotovora*; i) *E.c. carotovora*_SCC1 _which is a harpin (HrpN) producing strain [35], and ii) *E.c. carotovora*_SCC3193 _which is a HrpN-negative strain [36]. *B. cinerea *is a necrotrophic fungal pathogen that attacks over 200 different plant species [37], by producing multiple proteins and metabolites that kill the host cells [38]. The main virulence factors of *B. cinerea *vary depending on the isolate, and include toxins and cell wall degrading enzymes such as endopolygalacturonases and xylanases [39-41]. In this study, we demonstrate that *E.c. carotovora*-derived elicitors and *B. cinerea *cause disease symptoms and induce a defense response in the moss *Physcomitrella*.

## Results

### *E.c. carotovora *and *B. cinerea *infect *Physcomitrella patens*

**Figure 1:**
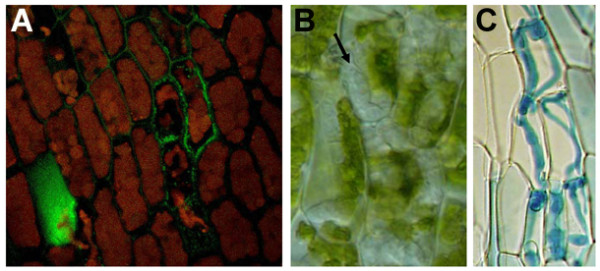
***E.c. carotovora *and *B. cinerea *inoculation of *Physcomitrella *leaves**. A. Leaves of *Physcomitrella *gametophores inoculated with *E.c. carotovora*_SCC3193 _carrying a GFP-expressing plasmid at 2 dpi. B. *B. cinerea *inoculated leaves at 2 dpi. C. Trypan blue stained *B. cinerea *hyphae in inoculated leaves at 2 dpi. Arrow indicates hyphae growing in *Physcomitrella*.

In order to determine whether *E.c. carotovora *infects *Physcomitrella *tissues, a gfp-labelled *E.c. carotovora*_SCC3193 _strain was inoculated onto *Physcomitrella *leaves as described in methods. Two days after inoculation tissue examined by confocal microscopy indicated that the labelled cells of *E.c. carotovora *had occupied the apoplast between leaf cells, as well as the cellular space of some plant cells in this same tissue (Figure 1A).

When *B. cinerea *inoculated leaf tissue was examined, outlines of fungal hyphae were apparent inside the cell cavity displacing the cytoplasmic contents (Figure 1B). Infection of *Physcomitrella *tissues by *B. cinerea *was examined in more detail by staining fungal hyphae with trypan blue. Two days post infection (dpi) hyphae appeared to be within the limits of the cell walls in *Physcomitrella *leaves (Figure 1C). Our observation that *B. cinerea *hyphae appeared within plant cells is likely in that *Physcomitrella *leaves are composed of a contiguous monolayer of adjacent cells.

### *E.c. carotovora*, CFs and *B. cinerea *cause disease symptoms in *Physcomitrella*

**Figure 2:**
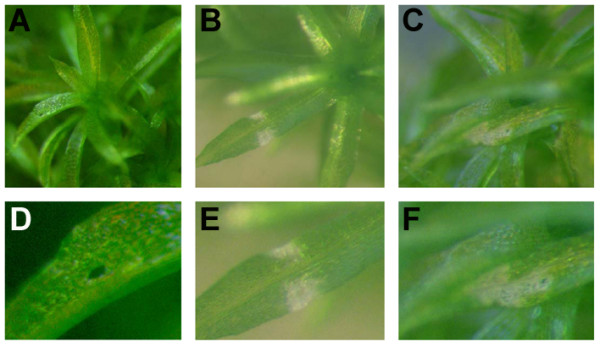
**Symptom development in response to *E.c. carotovora *inoculation**. Leaves of *Physcomitrella *gametophores were wounded and inoculated with 0.9% NaCl (A, D), *E.c. carotovora*_SCC3193 _(B, E) or *E.c. carotovora*_SCC1 _(C, F). Pictures of representative colonies were taken at 2 dpi.

Development of disease symptoms by *E.c. carotovora *was initiated by inoculating the harpin HrpN-producing *E.c. carotovora*_SCC1 _strain and the HrpN-negative *E.c. carotovora*_SCC3193 _strain onto *Physcomitrella *leaves. Inoculation with both strains caused visible symptoms around the wounded tissue within 2 days when observed with a magnifying glass, while mock inoculated tissues did not (Figure 2).

*Physcomitrella *infection by *E.c. carotovora *and subsequent development of symptoms required that we first wound the plant mechanically (Figures 1 and 2). Growth or colonization of *E.c. carotovora in planta *as determined by enumeration of bacteria in a given tissue could not be done due to the difficulty of consistently wounding the tissue sufficiently without causing excessive damage and dessication. Instead of continuing our studies with *E.c. carotovora *bacterial inoculation, cell-free culture filtrate (CF) was used to elicit a defense response since: i) in vascular plants CF incites the same disease symptoms and induces defense gene expression in the same way as does inoculation with *E.c. carotovora *[30-32], ii) CF is sprayed onto the colonies allowing for direct and homogeneous contact, and iii) it overcomes the technical difficulty of introducing a sufficient number of small wounds on the moss tissue to allow inoculation by *E.c. carotovora *for a comprehensive evaluation of the plant defense response.

**Figure 3:**
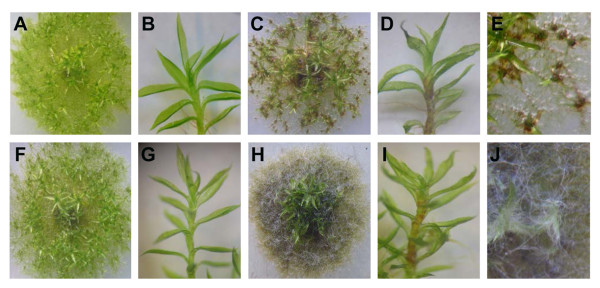
**Symptom development in response to CF treatment and *B. cinerea *inoculation**. Moss colonies and gametophores treated with LB (A, B), CF_(SCC1) _(C, D), CF_(SCC3193) _(F, G) or with *B. cinerea *spores (H, I). A closer view of colonies treated with CF_(SCC1) _(E), or inoculated with *B. cinerea *spores (J) is shown. Pictures of representative colonies were taken 2 days after treatment.

Disease symptom development was observed upon treating *Physcomitrella *colonies with: (i) CF of *E.c. carotovora*_SCC1 _(CF_(SCC1)_), (ii) CF of *E.c. carotovora*_SCC3193 _(CF_(SCC3193)_), or (iii) *B. cinerea *spores. Disease symptoms such as tissue maceration developed in colonies two days after treatment with either CF_(SCC1)_, CF_(SCC3193) _or after inoculation with *B. cinerea *spores, as shown in Figure 3. In control experiments, moss colonies treated with either Luria-Bertani (LB) (Figure 3A), potato dextrose broth (PBD) growth media (not shown) or with spores of a non-pathogen, *Aspergillus nidulans *(not shown), did not develop disease symptoms. Protonemal filaments treated with either CF_(SCC1) _or CF_(SCC3193) _developed maceration symptoms, with CF_(SCC3193) _causing less tissue damage than CF_(SCC1) _(Figures 3C and 3F). Additionally, CF_(SCC1) _-treated colonies acquired a brownish aspect as seen in the protonemal filaments shown in Figures 3C and 3E. CF-treated gametophores, also known as leafy shoots, did not show maceration symptoms, although brownish stems were observed in CF_(SCC1)_-treated colonies (Figure 3D).

*Physcomitrella *showed a clear susceptibility to *B. cinerea*, involving a characteristic proliferation of mycelium and the appearance of necrotic protonemal tissue in addition to the browning of stems (Figures 3H, 3J and 3I). Inoculated tissues were soft, macerated and were easily separated from the rest of the moss colony. Four dpi *B. cinerea*-infected moss tissues were completely macerated (data not shown). Protonemal filaments were more susceptible than leaves to CF treatments and *B. cinerea *inoculation. Taken together, these results show that CF_(SCC1)_, CF_(SCC3193) _and *B. cinerea *are capable of causing disease symptoms in *Physcomitrella*.

### CF_(SCC1) _and *B. cinerea *trigger cytoplasmic shrinkage, accumulation of autofluorescent compounds and chloroplast browning

**Figure 4:**
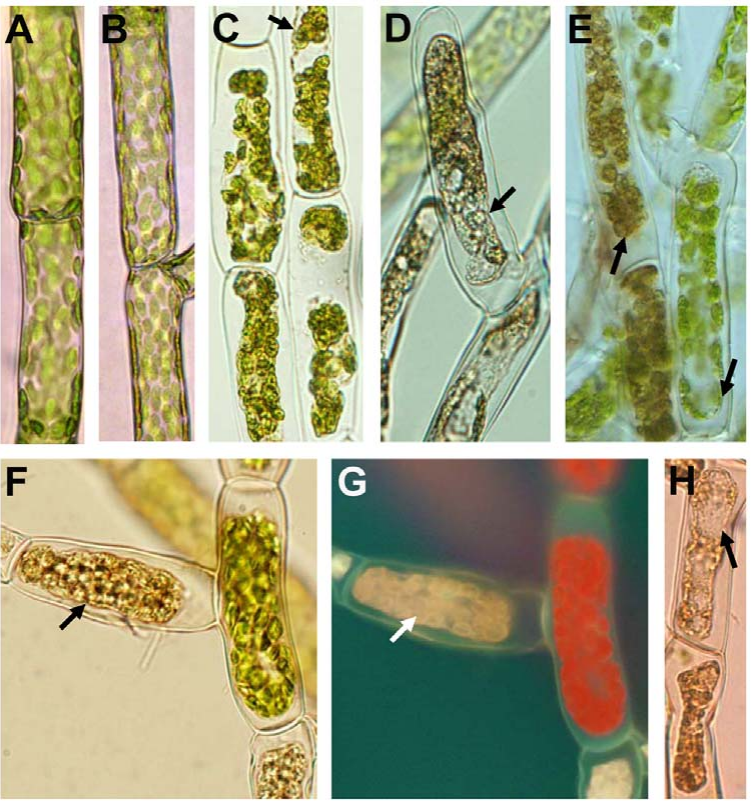
**Analysis of protonemal filament changes in response to treatments with CF and *B. cinerea *spores**. Protonemal filaments examined under transmitted light after 2 days of treatment with LB (A), CF_(SCC3193) _(B), CF_(SCC1) _(C, D), and *B. cinerea *spores (E). Cytoplasmic shrinkage observed with CF_(SCC1) _and *B. cinerea *spores are indicated with arrows. CF_(SCC1)_-treated protonemal cells showing browning of chloroplasts and loss of red chlorophyll autofluorescence after UV-excitation are indicated with arrows (F, G). A cell with collapsed cytoplasm and fewer chloroplasts is shown 4 days after treatment (indicated with an arrow) (H).

Pathogen infection or elicitor treatment can induce plant cell death with characteristic changes in cells, including cytoplasmic shrinkage, alteration of chloroplast organization and accumulation of autofluorescent compounds [13,42-44]. In the present study, we examined cellular changes occurring in *Physcomitrella *tissue showing macroscopic disease symptoms after exposure to *B. cinerea *spores or CF of the *E.c. carotovora *strains. CF_(SCC1)_-treated (Figures 4C and 4D) and *B. cinerea*-inoculated (Figure 4E) protonemal cells showed cytoplasmic shrinkage after 2 days. In contrast, no cytoplasmic shrinkage was evident in cells treated with LB (control for CF treatments, Figure 4A), CF_(SCC3193) _(Figure 4B) or PDB (control for *B. cinerea *inoculation, data not shown). Other morphological changes were also observed in *Physcomitrella *CF_(SCC1)_-treated and *B. cinerea*-inoculated cells. After 2 days, both treatments caused browning of the chloroplasts in a high proportion of cells (Figures 4D and 4E). Chloroplast browning was evident only in cells showing cytoplasmic shrinkage (Figures 4D and 4E). Additionally, CF_(SCC1)_-treated (Figures 4F and 4G) and *B. cinerea*-inoculated cells (data not shown) with brownish chloroplasts showed lack of red autofluorescence of chlorophyll. CF_(SCC1)_-treated protonemal cells having cytoplasmic shrinkage and brown chloroplasts were more abundant after 4 days. Within this treated protonemal tissue, cells with fewer brown chloroplasts were also observed suggesting that they were being brokendown (Figure 4H). In contrast, CF_(SCC3193)_- or LB-treated protonemal filaments did not show such changes and green chloroplasts were evident at least 6 days after treatment (data not shown).

**Figure 5:**
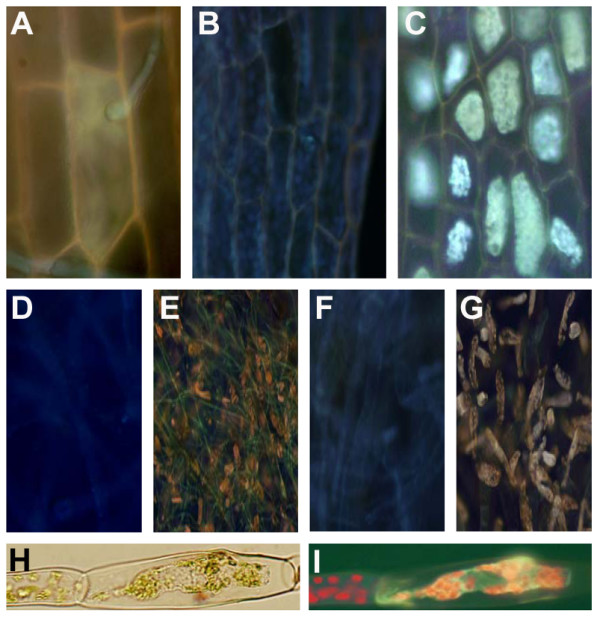
**Accumulation of autofluorescent compounds in *Physcomitrella *after CF treatment and *B. cinerea *inoculation**. Examination of UV-stimulated autofluorescence of *B. cinerea*-inoculated leaf (A, C), PDB-treated leaf (B), PDB- (D), *B. cinerea *spores- (E), LB- (F) and CF_(SCC1)_-treated protonemal filaments (G). A closer view of a CF_(SCC1)_-treated protonemal cell with cytoplasmic shrinkage and UV-stimulated autofluorescence is shown (H, I). Observations were made 2 days after treatments.

After an apparent initial contact of *B. cinerea *hyphae with individual cells within the leaf, plant cells were observed to respond by accumulating autofluorescent compounds (Figure 5A). Cells in *B. cinerea*-inoculated leaves developing light blue to yellow autofluorescence (AF) were also observed. This AF appeared confined to the cytoplasm now separated from the cell wall (Figure 5C). No AF, however, was observed in CF_(SCC1)_- or CF_(SCC3193)_-treated leaves (data not shown). AF was clearly evident in protonemal filaments of *B. cinerea*-inoculated and CF_(SCC1)_-treated colonies (Figures 5E and 5G). In contrast, no AF was seen in PDB-treated leaves (Figure 5B) or PDB- or LB-treated protonemal filaments (Figures 5D and 5F), and only a few CF_(SCC3193)_-treated cells accumulated autofluorescent compounds (data not shown). Also, accumulation of autofluorescent compounds was generally observed once cytoplasmic shrinkage occurred (Figures 5H and 5I). In summary, our results show that CF_(SCC1) _and *B. cinerea *induce cellular changes in *Physcomitrella *protonemal cells, including cytoplasmic shrinkage, browning of chloroplasts and accumulation of autofluorescent compounds, suggesting a cell death process.

### *E.c. carotovora *elicitors and *B. cinerea *trigger cell death in *Physcomitrella*

**Figure 6:**
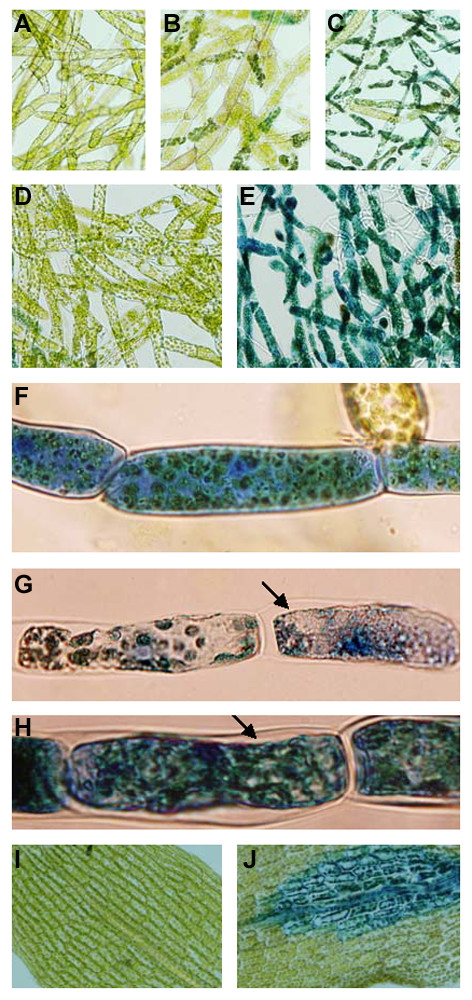
**Analysis of cell death in *Physcomitrella***. Evans blue staining of protonemal tissues after treatments with LB (A), CF_(SCC3193) _(B), CF_(SCC1) _(C), PDB (D) and *B. cinerea *spores (E). A closer view of CF_(SCC3193)_- (F), CF_(SCC1)_- (G) and *B. cinerea *inoculated (H) protonemal cells is shown. Arrows indicate cytoplasmic shrinkage. Leaves treated with CF_(SCC1) _(I) and *B. cinerea *spores (J) were also stained with Evans blue. Pictures of representative tissues were taken 2 days after treatment.

To assess whether CF and *B. cinerea *caused cell death of *Physcomitrella *tissues, we stained moss colonies with Evans blue, a dye that is excluded by membranes of living cells but diffuses into dead cells [45]. Figure 6 shows pictures of representative tissues. Two days after treatments, an increase in stained cells was observed with either CF_(SCC3193)_, CF_(SCC1) _or *B. cinerea *spores, compared with control treatments. Although, while in CF_(SCC3193)_-treated tissue a low proportion of stained protonemal cells was observed (Figure 6B), CF_(SCC1)_-treated or *B. cinerea*-inoculated tissues showed a high proportion of stained protonemal cells (Figures 6C and 6E). In control treatments, almost no stained cells were visible (Figures 6A, 6D).

Cytoplasmic shrinkage was evident in most Evans blue stained protonemal cells treated with CF_(SCC1) _or *B. cinerea *spores (Figures 6G and 6H). Whenever cytoplasmic shrinkage occurred, cells were stained with Evans blue, indicating that these cells were dying or dead. In contrast, most CF_(SCC3193)_-treated protonemal cells did not show cytoplasmic shrinkage and the dye was distributed homogeneously in the cells (Figure 6F). Three days after treatment, stained CF_(SCC3193)_-treated protonemal cells did not exhibit cytoplasmic shrinkage suggesting that this response does not develop at a later stage (data not shown). In gametophores, Evans blue stained cells could be detected in leaves inoculated with *B. cinerea *(Figure 6J), whereas stained cells were not seen after CF_(SCC1) _(Figure 6I), CF_(SCC3193) _or control treatments (not shown).

### *B. cinerea *and *E.c. carotovora *elicitors mediate activation of defense-related genes

**Figure 7:**
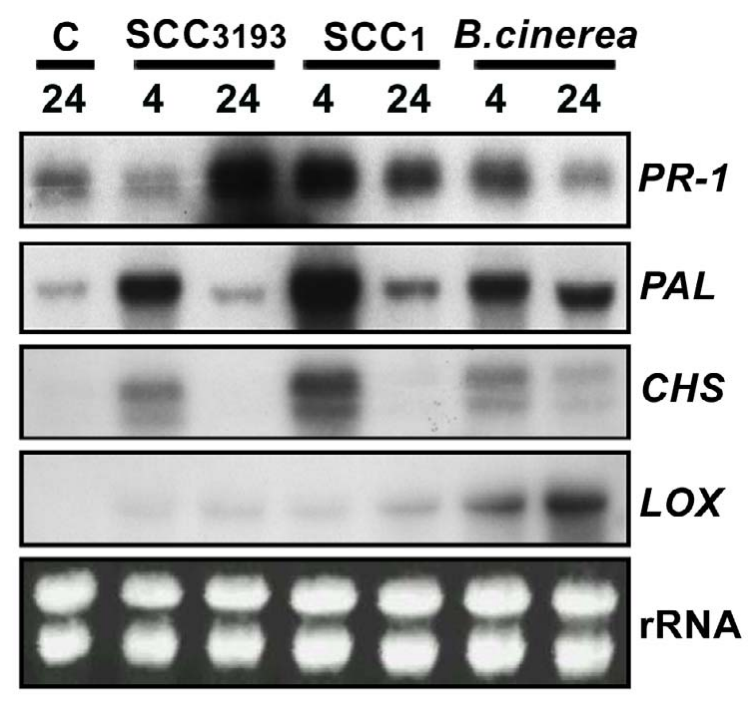
**CF and *B. cinerea*-induced expression of defense-related genes in *Physcomitrella***. Expression of *PR-1*, *PAL*, *CHS *and *LOX *genes was characterized by RNA-gel blot hybridization after the following treatments: moss colonies sprayed with LB (control, C), CF_(SCC1)_, CF_(SCC3193) _or inoculated with *B. cinerea *spores (2 × 10^5 ^spores/ml). Plant samples were harvested at the indicated times (hours) after treatment. 10 μg of RNA was separated on formaldehyde-agarose gels, transferred to nylon membranes and hybridized to ^32^P-labeled DNA probes. Ethidium bromide staining of rRNA was used to ensure equal loading of RNA samples. Similar results were obtained from two independent experiments.

To analyze whether CF treatment or *B. cinerea *inoculation trigger *Physcomitrella *defense gene expression, we characterized the expression of a number of defense-related gene homologues including; (i) *PR-1*, (ii) *LOX*, (iii) *PAL*, and (iv) *CHS*. LOX (lipoxygenase) is a key enzyme in the synthesis of defense-related compounds including JA [46], PAL (phenylalanine ammonia-lyase) mediates the biosynthesis of phenylpropanoids and SA [5,47] and CHS (chalcone synthase) is the first enzyme in the synthesis of flavonoids [5]. The results in Figure 7 show that expression of the *Physcomitrella *homologues increased after CF treatment or *B. cinerea *inoculation. Clearly, three types of expression patterns were observed. The level of *PR-1 *expression peaked at 24 h in CF_(SCC3193)_-treated moss colonies, while in CF_(SCC1)_-treated and *B. cinerea*-inoculated tissues expression of *PR-1 *peaked at 4 h. Expression of *PAL *and *CHS *peaked at 4 h in tissues treated with either of the CFs or with *B. cinerea *spores, although among the treatments, higher expression levels were observed with CF_(SCC1)_. In the case of *CHS*, two transcripts with an identical expression pattern were detected. *LOX *expression was moderately induced in CF_(SCC1)_- and CF_(SCC3193)_-treated moss colonies at 4 and 24 h, while transcript levels increased significantly in *B. cinerea *inoculated *Physcomitrella *tissues, reaching the highest expression level at 24 h. The results obtained in this study show that several conserved defense-related gene homologues of *Physcomitrella *were induced in response to treatment with *E.c. carotovora *elicitors or *B. cinerea *spores.

## Discussion

The developmental simplicity, ease of genetic analysis and the evolutionarily relationship between *Physcomitrella *and other plants has prompted us to study the interaction between this moss and two broad host range pathogens, the bacterium *E.c. carotovora *and the fungus *B. cinerea*. Our results indicate that both *B. cinerea *and *E.c. carotovora *can infect *Physcomitrella *tissues and cause disease symptoms. Since *Physcomitrella *gametophytes do not have stomata, *E.c. carotovora *entered plant tissues through wounds, while *B. cinerea *hyphae probably entered by hydrolyzing the plant cell wall using hydrolytic enzymes or by secreting cell wall permeable toxins that kill plant cells. We have observed *B. cinerea *hyphae within plant cells, including cells in which hyphae were apparently inside the cell cavity displacing the cytoplasm. Hyphae of other necrotrophic fungi, including *S. sphagnicola*, *Tephrocybe palustris *and *Nectria mnii*, are capable of penetrating live cells of moss leaves, resulting in cell death *a posteriori *[23,48]. In case of *Nectria mnii*, it was shown that intracellular hyphae could displace the host cell contents [48].

*E.c. carotovora*_SCC1_, but not *E.c. carotovora*_SCC3193_, was previously shown to harbour the harpin-encoding gene *hrpN *[35,36]. Harpins are bacterial effector proteins released into the host cells, through a type III secretion system encoded by the hypersensitive reaction and pathogenicity (*hrp*) gene cluster. When present in plant tissue, harpins cause HR and induction of defense mechanisms [49,50]. In *Erwinia *spp. *hrp *genes have been shown to contribute to virulence and to the ability of the pathogen to grow in the plant [35,51]. The higher maceration rate of the protonemal tissues observed with CF_(SCC1) _compared with CF_(SCC3193) _is consistent with previous studies showing that polygalacturonase, together with harpin HrpN from *E.c. carotovora*_SCC1 _greatly enhanced lesion formation in *Arabidopsis *[52].

Cytoplasmic shrinkage is the most common morphological change occurring in plant PCD and has been observed in cells undergoing HR, as well as in tissues of plants susceptible to virulent pathogens [53-55]. Cytoplasmic shrinkage was observed only in Evans blue stained protonemal cells treated with CF_(SCC1) _but not with CF_(SCC3193)_, probably suggesting that a different mechanism leading to cell death had occurred. This finding is consistent with the induction of HR by harpins and with previous results showing that *Pseudomonas syringae *pv *phaseolicola *induced cytoplasmic shrinkage in plant cells, while a *hrpD *mutant did not [43].

Breakdown of chloroplast membranes and chlorophyll has been observed in cells undergoing PCD, including those treated with elicitors, infected with pathogens or those undergoing senescence [56-59]. Our results showed that treatments with CF_(SCC1) _and *B. cinerea *spores induce browning of chloroplasts, which is likely followed by the breakdown of these organelles. This is consistent with previous results showing that *S. sphagnicola *hyphae are capable of causing degeneration of chloroplasts in the moss *S. fuscum *[23]. Chloroplasts remained green in protonemal CF_(SCC3193)_-treated cells, while boiled CF_(SCC1)_, containing the heat-stable HrpN, still induced browning of the chloroplasts in cells also showing cytoplasmic shrinkage (data not shown). These findings suggest that HrpN might trigger cell death associated with cytoplasmic shrinkage and chloroplasts browning. In addition, browning of chloroplasts was associated with chlorophyll breakdown in CF_(SCC1)_-treated and *B. cinerea*-inoculated cells, since no red chlorophyll autofluorescence was observed (although quenching by other compounds cannot be excluded). These results are supported by findings showing that *E.c. carotovora*_SCC1 _induced the expression of chlorophyllase 1 in *Arabidopsis*, which could be involved in the degradation of photoactive chlorophylls to avoid higher levels of reactive oxygen species (ROS) production and cellular damage during pathogen infection [60]. It is also interesting to note that whenever browning of chloroplasts was observed in CF_(SCC1)_-treated or *B. cinerea*-inoculated cells, cytoplasmic shrinkage was also present. Since CF_(SCC1)_-treated protonemal cells with cytoplasmic shrinkage but green chloroplasts were also observed, browning of chloroplasts could be a process occurring later in dying cells after collapse of the cytoplasm. Browning of chloroplasts could be indicative of oxidative processes due to excessive accumulation of ROS in the chloroplasts at late stages of CF_(SCC1) _and *B. cinerea *treatments, finally leading to chloroplasts breakdown. To our knowledge, this is the first report in which browning of chloroplasts was observed after pathogen and elicitor treatment. The ability to observe changes in the coloration of the chloroplasts was facilitated in that the leaf tissue, like the protonemal filaments, is composed of a single monolayer of cells.

Accumulation of autofluorescent compounds has been associated with the occurrence of HR in vascular plants [6,44]. CF_(SCC1)_-treated or *B. cinerea*-inoculated *Physcomitrella *tissues developed AF. A previous report demonstrated localized deposition of phenolic compounds at the sites of fungal penetration and also as a second major response that appeared to follow cell death [44]. These findings are consistent with our results, in showing AF confined to the collapsed cytoplasm of dead cells in *B. cinerea*-inoculated leaves and protonemal filaments.

*B. cinerea *induce PCD to enable rapid colonization of vascular plants, and *Erwinia *harpins have been shown to elicit cell death [49,50,61,38]. Cytoplasmic shrinkage, an indicator of plant PCD, correlated with accumulation of autofluorescent compounds and chloroplast browning after inoculation with *B. cinerea *or treatment with CF of HrpN producing *E.c. carotovora*_SCC1 _suggesting that either treatment results in PCD in *Physcomitrella*.

Our results also showed that *E.c. carotovora *elicitors and *B. cinerea *induced defense-related gene expression in *Physcomitrella*. Earlier induction of the *PR-1*-like gene expression and the higher levels of *PAL *and *CHS *mRNA accumulation triggered by CF_(SCC1) _compared with CF_(SCC3193)_, corresponded well with the higher levels of tissue maceration observed with CF_(SCC1)_. *CHSs *are encoded by multiple genes in vascular plants and *Physcomitrella *[5,62], and in our study two *CHS *transcripts with an identical expression pattern were detected. Recently, a new enzymatic activity was described for the same *Physcomitrella *LOX gene product induced by *B. cinerea *in this study [63]. Novel oxylipins were generated by this enzyme suggesting a possible involvement in defense responses. In vascular plants *PR-1*, *PAL *and *LOX *are induced by inoculation with *E.c. carotovora *or by CF treatments [52,64,65] and *PR-1 *transcript accumulation is increased after *B. cinerea *infection [66,67]. The results obtained in this study suggest that *E.c. carotovora *elicitors and *B. cinerea *similarly induce expression of *Physcomitrella *defense gene homologues of those studied in vascular plants, and thus validate the use of non-specific plant pathogens or elicitors derived from them to study moss-pathogen interactions.

## Conclusion

In the present study, we demonstrate that *E.c. carotovora *elicitor treatment and *B. cinerea *inoculation cause disease symptoms and induce defense responses in *Physcomitrella*. CF_(SCC1)_, CF_(SCC3193) _and *B. cinerea *induced the expression of defense-related genes, including *PR-1*, *LOX, PAL *and *CHS *homologues. Compounds produced by LOX, PAL and CHS are involved in the synthesis of JA, phenylpropanoids and SA and flavonoids, respectively, in vascular plants. These compounds could play a role in the defense response of *Physcomitrella *as has been shown in vascular plants. As such our results further establish *E.c. carotovora *elicitors, as well as *B. cinerea *as promising systems to analyze induction of defense responses in *Physcomitrella*.

Since cytoplasmic shrinkage is the most common morphological change observed in plant PCD, and that harpins and *B. cinerea *induce this type of cell death in vascular plants, our results suggest that *E.c. carotovora *CF_SCC1 _containing HrpN and *B. cinerea *could also induce this type of cell death in *Physcomitrella*. Finally, the occurrence of distinct cellular responses leading to cell death by CF_(SCC1) _and CF_(SCC3193) _provides a useful system to analyze pathogen-induced cell death and to characterize the key elements involved in its regulation by targeted gene disruption in *Physcomitrella*.

## Methods

### Plant material and growth conditions

*Physcomitrella patens *Gransden WT isolate [68] was grown on cellophane overlaid BCDAT agar medium consisting of 1.6 g l ^-1 ^Hoagland's, 1 mM MgSO_4_, 1.8 mM KH_2_PO_4 _pH 6.5, 10 mM KNO_3_, 45 μM FeSO_4_, 1 mM CaCl_2_, 5 mM ammonium tartrate and 10 g l^-1 ^agar [69]. Protonemal cultures and moss colonies were grown as described previously [70]. Plants were grown at 22°C under a photoperiod of 16 h light and three-week-old colonies were used for all the experiments.

### Pathogen inoculation and culture filtrate treatments

*Erwinia carotovora *ssp *carotovora *strains SCC3193 [71] and SCC1 [72] were propagated on LB medium [73] at 28°C. Cell-free culture filtrates were prepared by growing bacteria in LB broth overnight, removing bacterial cells by centrifugation (10 min at 4000 g) and filter sterilizing the supernatant (0.2 μm pore size). This filter-sterilized supernatant (CF) was applied by spraying the moss colonies (3 ml per Petri dish containing 16 moss colonies). *E.c. carotovora*_SCC3193 _and *E.c. carotovora*_SCC1 _were grown on LB, and *E.c. carotovora*_SCC3193 _transformed with plasmid pUC18 containing the GFP sequence as reporter gene under control of the *lac *promoter was grown on LB containing 100 μg/ml ampicillin. After 16 h bacterial cells were centrifuged and suspended in 0.9% NaCl to a final concentration of 5 × 10^8 ^cfu/ml. These suspensions were used for inoculation of *Physcomitrella *leaves previously wounded with a needle to create small lesions. An isolate of *B. cinerea *from a lemon plant was cultivated on 39 g/L potato dextrose agar (DIFCO) at room temperature. *B. cinerea *was inoculated by spraying a 2 × 10^5 ^spores/ml suspension in half-strength PDB (DIFCO). Symptom development of CF-treated and *B. cinerea*-inoculated *Physcomitrella *colonies was analyzed in three independent experiments using two Petri dishes containing16 colonies each. The experiments involving leaves inoculated with *E.c. coratovora *strains SCC1, SCC3193 and SCC3193 carrying the GFP marker were performed at least three times.

### Evans blue and trypan blue staining, autofluorescence detection and microscopy

For detection of cell death, moss colonies were incubated for 2 hours with 0.05% Evans blue and washed 4 times with deionized water to remove excess and unbound dye. Growth and development of *B. cinerea *mycelium inside leaf tissues was monitored by staining with lactophenol-trypan blue and destaining in saturated chloral hydrate as described previously [74]. For autofluorescent compound detection, leaves were boiled in alcoholic lactophenol and rinsed in ethanol and water [75]. Material was then mounted on a slide in 50% glycerol and examined for Evans blue or trypan blue staining or using ultraviolet epifluorescence for detection of autofluorescent compounds (Microscope Olympus BX61). The infection of *Physcomitrella *leaves by GFP-tagged *E.c. carotovora *was visualized with a laser scanning confocal microscope FV 300 (Olympus).

### RNA gel blot analysis

Total RNA was isolated from control and treated plant tissue corresponding to 64 moss colonies, using standard procedures based on phenol/chloroform extraction followed by LiCl precipitation. Ten micrograms of total RNA separated by denaturing agarose-formaldehyde gels was transferred to a nylon membrane (Hybond N) following standard procedures [76]. Membranes were prehybridized at 65°C in 6 × SCC, 0.5% SDS, 0.125 mg milk powder and 0.5 mg ml^-1 ^denatured salmon sperm DNA. Hybridizations were performed at 65°C overnight. The DNA fragments to be used as probes were obtained by PCR using the plasmid harbouring the corresponding cDNA as template and the primers M13 forward and reverse. The cDNA clones used were: [DDBJ:BJ182301 (*PR-1*), DDBJ:BJ201257 (*PAL*), DDBJ:BJ192161 (*CHS*) and DDBJ:BJ159508 (*LOX*)]. PCR fragments were purified using Qiaquick columns (Qiagen), and were labelled with [α^32^P]-dCTP using Rediprime II Random Prime labelling system (Amersham Biosciences). After hybridization, membranes were washed twice for 30 min at 65°C with 5 × SCC, 0.1% SDS and twice 30 min with 2 × SCC, 0.1% SDS. Subsequently, membranes were exposed on autoradiography film. The amount of RNA loaded was verified by addition of ethidium bromide to the samples and photography under UV light after electrophoresis.

## Authors' contributions

IPDL participated in the Northern blot analysis and the microscopic studies, designed this study, drafted and edited the manuscript. JPO carried out the analysis of symptom development and all the microscopic studies. AC and MB participated in the cell death analysis by Evans blue staining. CG participated in the Northern blot analysis. SV helped to draft the manuscript. All authors read and approved the final manuscript.
